# Daptomycin-Induced Hyperkalemia as an Early Sign of Rhabdomyolysis in a Diabetic Patient

**DOI:** 10.7759/cureus.11674

**Published:** 2020-11-24

**Authors:** Gabriel Ibarra, Ahmed Elmaaz, Danielle Hennel, Enrique Pacheco

**Affiliations:** 1 Internal Medicine Department, New York Medical College, Metropolitan Hospital Center, New York, USA; 2 Physician Assistant Program, Touro College of Osteopathic Medicine, New York, USA; 3 Internal Medicine Department, Yale University, Waterbury, USA

**Keywords:** antibiotics, daptomycin, hyperkalemia, rhabdomyolysis, methicillin resistant staphylococcus aureus (mrsa), vre, cpk

## Abstract

Daptomycin is a lipopeptide antibiotic that is active against vancomycin-resistant enterococci (VRE) and methicillin-resistant *Staphylococcus aureus* (MRSA). It is less nephrotoxic than vancomycin. It has a unique bactericidal mechanism through destruction of bacterial membrane potential. However, one of the most clinically relevant adverse effects of daptomycin is reversible myopathy, especially when daptomycin is used in high doses. Here, we present a case of a patient with rhabdomyolysis preceded by hyperkalemia associated with daptomycin use. Soon after daptomycin administration, hyperkalemia was noticed before the acute rise in creatinine phosphokinase (CPK). The serum levels of potassium and CPK returned to normal after daptomycin was stopped which suggested the causal relationship between hyperkalemia and myopathy and daptomycin use. To our knowledge, this is the second case of hyperkalemia related to daptomycin use.

## Introduction

Daptomycin is a lipopeptide antibiotic that has recently stood in the frontline as a good alternative for the management of methicillin-resistant *Staphylococcus aureus* (MRSA) and *Enterococcus* species infections [1]. Despite the good coverage against these microorganisms, daptomycin, when used, has been found to be associated with increased risk of rhabdomyolysis. Hyperkalemia is not enlisted as one of the adverse effects caused by daptomycin. Here, we present a case of a diabetic patient with recurrent elevation in serum potassium level after daptomycin initiation and prior to recorded creatinine phosphokinase (CPK) elevation with normalization of serum potassium and CPK levels once daptomycin was discontinued.

## Case presentation

**Figure 1:**
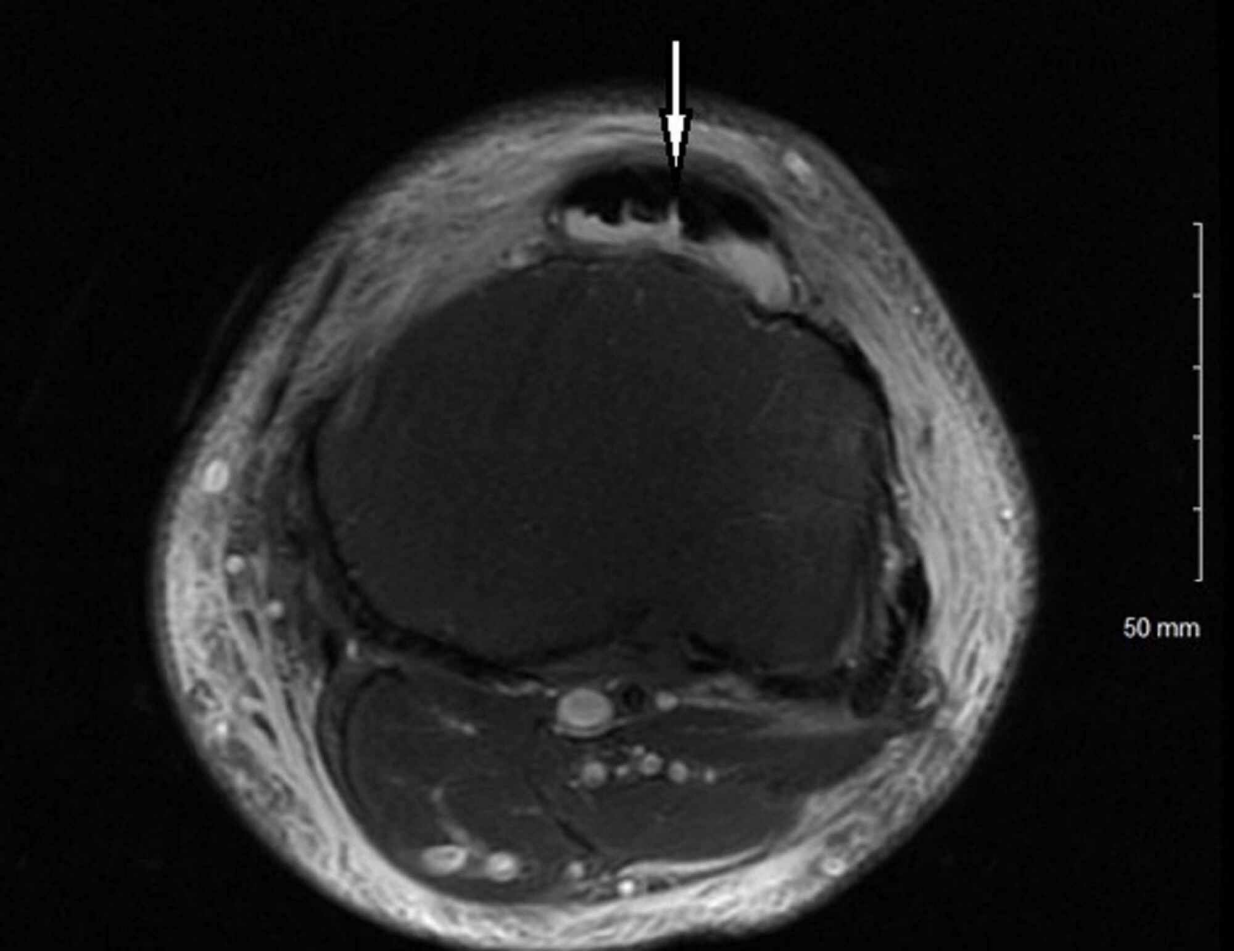
Severe subcutaneous edema and skin thickening compatible with cellulitis. 4.2 x 3.3 cm complex fluid loculation in the subcutaneous soft tissue anterior to the patella representing prepatellar bursitis with fluid loculation in the infrapatellar fat pad (white arrow).

A 42-year-old male with a past medical history of Type II diabetes mellitus presented to the emergency department with a chief complaint of left knee pain starting one week prior. Vital signs on admission showed a blood pressure of 97/66 mmHg and a heart rate of 100 beats per minute. The initial body temperature was 98.7 degrees F followed by a subsequent rise to 102.8 degrees F after 24 hours. The physical exam was remarkable for left knee edema and erythema with a 5 cm lump in the prepatellar region that was warm and tender to palpation. Prior to presentation, the patient was on metformin 1 gram every 12 hours but was non-adherent. He was not taking any renin-angiotensin-aldosterone system (RAAS) inhibitors or statin therapy. Initial workup revealed serum creatinine level of 0.9 mg/dL and blood urea nitrogen (BUN) level of 19 mg/dL associated with a blood glucose of 610 mg/dL, a bicarbonate level of 24 mEq/L, hyponatremia at 126 mEq/L (corrected sodium of 134 mEq/L) with anion gap of 12. Potassium was 4.7 mEq/L. Magnetic resonance imaging (MRI) of the left knee showed pre-patellar abscess with bursitis (Figure 1).

**Figure 2:**
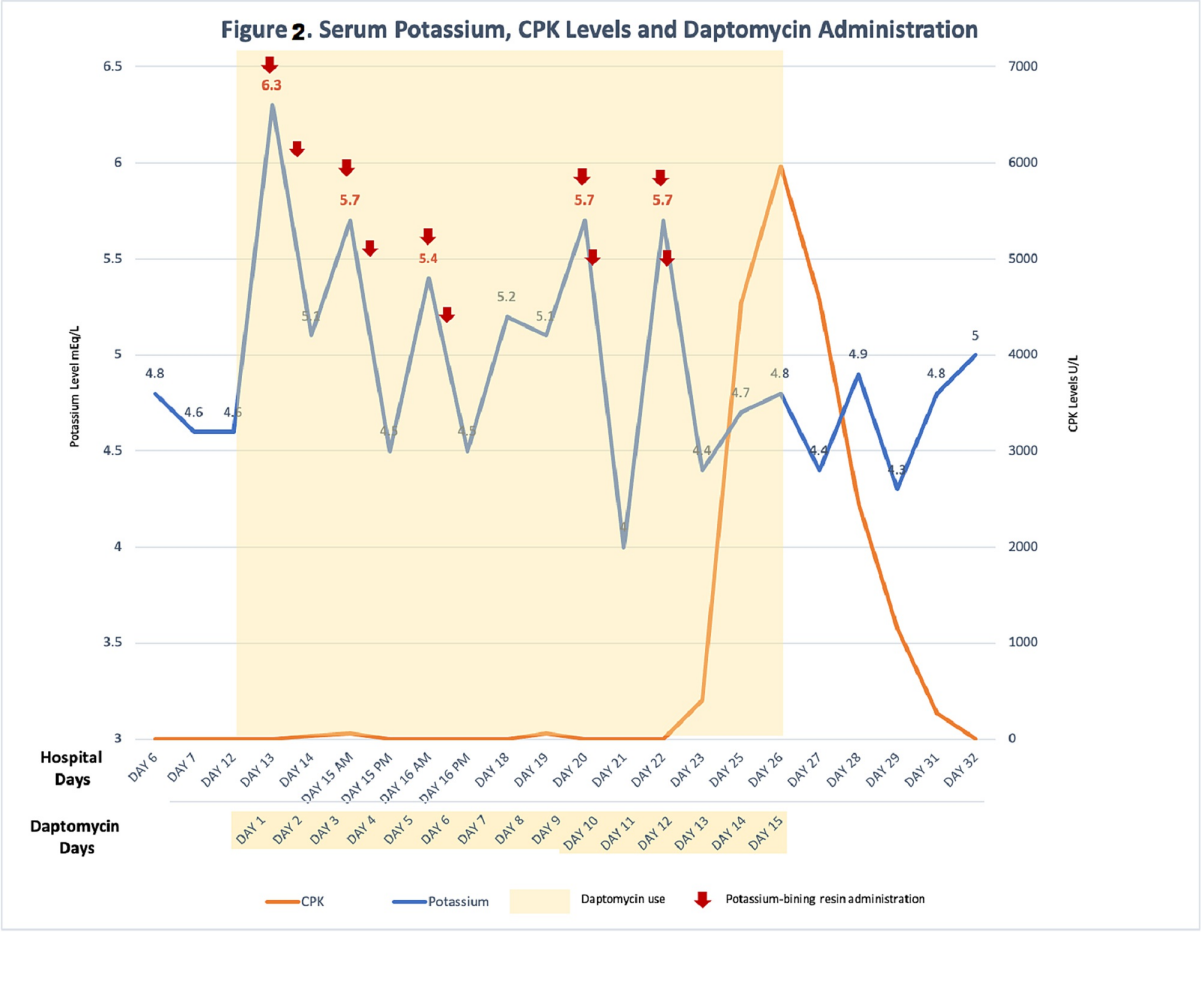
Serum potassium changes associated with daptomycin use. Note that after each provision of potassium-binding resin, a decrease in serum potassium was recorded (Red arrows). After every challenge with daptomycin from day 12 to day 22 (yellow background), serum potassium increased on six occasions with five out of them are in the range of hyperkalemia. Eleven days after initiation of daptomycin and recurrent hyperkalemia, creatinine phosphokinase (CPK) spiked to 4,537 mg/dL (orange line).

The patient was hospitalized for septic bursitis and intravenous fluids with empirical antibiotic therapy of vancomycin and piperacillin-tazobactam at the doses of 1.25 grams every 12 hours and 4.5 grams every six hours respectively, were initiated. Drainage of the abscess was performed with an elevated count of polymorphonuclear leucocytes (PMN). The tissue culture was negative for any microorganism growth. Increase of serum creatinine and BUN levels to 1.4 mg/dL and 29 mg/dL, respectively, occurred on day three of hospitalization suggesting acute kidney injury, which normalized after intravenous fluids. Infectious diseases service was consulted on day 12 of hospitalization given frequent out of range serum levels of vancomycin trough and concerns of the patient regarding repeated monitoring of blood level of vancomycin. Therefore, it was recommended to switch vancomycin to daptomycin 400 mg IV daily. To that point, laboratory findings did not show a single episode of hyperkalemia. On day 13 of hospitalization, 24 hours after daptomycin was started, the serum level of potassium increased to 6.3 mEq/L without electrocardiographic changes. Oral binding resin was given with a subsequent decrease of potassium levels to 5.1 mEq/L 12 hours later (Figure 2).

Common causes of isolated hyperkalemia were considered. Among them, heparin was discontinued and tighter finger stick glucose controls were made with a goal of <180 mg/dL. Potassium levels were again registered elevated on the second and third day (days 10 and 11 after hospitalization) after daptomycin was initiated (Figure 2). Aldosterone level of 4.8 ng/dL (reference range: <23.2 ng/dL), a renin level of 0.732 ng/mL/hour (reference range 0.167-5.380 ng/mL/hour) and a fraction excretion of urine sodium (FeNa) calculated of <1% with normal chloride levels of 102 mEq/L excluded the possibility of renal tubular acidosis (RTA) type 4. Plasma potassium level was started to be measured instead of serum potassium to rule out pseudo-hyperkalemia caused by laboratorial artifact.

Daily potassium monitoring showed again an increment of potassium level after daptomycin infusion on three more occasions and the patient was again treated with oral binding resin three more times (Figure 2). On day 14 after daptomycin initiation, CPK had a significant increase from the normal range measured two days prior to 4,537 mg/dL and the diagnosis of rhabdomyolysis was made.

Algorithms like the World Health Organization-Uppsala Monitoring Centre (WHO-UMC) system and the Naranjo algorithm are commonly used to assess the likelihood that an adverse reaction was caused by a medication. We used the Naranjo algorithm, which is less prone to subjective variations compared to other systems [2]. The modified Naranjo algorithm asks 10 questions related to the probability for a drug to be the cause of the adverse reaction and provides a maximum score of 13 points. Scores from 1 to 4 points make the reaction possible, from 5 to 8 points probable, and above 9 points make it definite. Based on the Naranjo likelihood algorithm, it was probable that daptomycin could be the main factor.

Daptomycin was discontinued and vancomycin was reinitiated. Once daptomycin was discontinued and intravenous fluids were provided, no new potassium derangements were registered throughout the rest of his hospital stay and CPK levels returned to normal levels with no further elevations. The patient completed a four-week-course of antibiotics with vancomycin and cefepime. No new episodes of hyperkalemia were registered and the patient was discharged home without complications.

## Discussion

Daptomycin is a bactericidal lipopeptide that could be a reasonable alternative for treating gram-positive articular and joint infections when vancomycin is not well-tolerated [1].

Despite the good coverage for these microorganisms, rhabdomyolysis is considered the main adverse effect of daptomycin use. Rhabdomyolysis is more commonly seen in a dose-dependent manner, especially at higher doses of daptomycin (8 mg/kg/day) compared to standard doses (6 mg/kg/day) [3]. Here, we described a case of recurrent hyperkalemia when daptomycin was initiated and completely resolved once daptomycin was discontinued.

Common medications such as angiotensin receptor blockers (ARBs), angiotensin-converting enzyme inhibitors (ACEi), and Trimethoprim-Sulfamethoxazole (TMP-SMX) are associated with hyperkalemia in their adverse effect list and are needed to be considered as potential causes of elevated serum potassium when a differential diagnosis is considered. Hyperkalemia is not listed as one of the adverse effects caused by daptomycin.

To our knowledge, this is the second case report of hyperkalemia associated with daptomycin use after Budovich et al. [4]. In their case report, daptomycin was used at a dose of 9 mg/kg/day, the same dose used in our case. Hyperkalemia was recorded on day 14 of hospitalization, with normalization of potassium level upon temporary withholding of daptomycin for one day and the use of potassium binding resin. That was followed by recurrence of hyperkalemia with restarting of daptomycin even at a lower dose (7 mg/kg), which supported the temporal relationship between daptomycin use and hyperkalemia. It is worth noting that the use of lower doses of daptomycin was associated with milder elevations of serum potassium levels. Additionally, despite the absence of evidence of heparin discontinuation, they mentioned that on days 14 and 15 of hospitalization, there was no documentation of the scheduled venous thromboembolism prophylaxis. Interestingly, they described an elevation of CPK concentration of 6.7 times the upper limit of normal on day 10 of hospitalization, which was four days prior to the first recorded episode of hyperkalemia, followed by a later decline of CPK concentration to twice the upper limit of normal by day 15. Of importance, the serum concentration of potassium was not recorded on days 11 and 12 of hospitalization in their patient, and on day 13, potassium level was 5.4 mEq/L, which was the upper limit of normal. 

A limited amount of literature highlights the relationship between hyperkalemia and daptomycin use. Claeys et al. reported hyperkalemia in 10 (35.7%) out of 28 patients who received a combination of daptomycin and TMP-SMX [5]. The overall median level of potassium was 5.1 mEq/L. However, among those with reported elevated level of potassium, the median potassium level was 5.8 mEq/L. The highest levels of serum potassium, more than 6 mEq/L, were seen in patients with chronic kidney disease on renal replacement therapy. Although TMP-SMX per se has been associated with hyperkalemia, the combination of both TMP-SMX with a high dose of daptomycin may increase the risk of hyperkalemia. Importantly, Gentry et al. also reported an increased incidence of hyperkalemia associated with a high dose of TMP-SMX, and with concomitant use of medications that can increase creatinine or potassium levels, such as RAAS inhibitors. Therefore, daptomycin should be used cautiously along with other hyperkalemia causing medications [6]. 

Daptomycin-induced myopathy has been described as a reversible skeletal-muscle myopathy [7]. The molecular mechanism of CPK release from rhabdomyocytes has been hypothesized to be mediated by membrane pore-like formation by daptomycin polymers intertwining with lipids in the rhabdomyocyte membrane [7]. The bactericidal mechanism of action of daptomycin is by forming complexes with calcium and phosphatidylglycerol (PG), creating pore-channels selective for cations, such as Na+, K+, and other alkali metal ions [8]. Because the membranes of the human cells, including muscle cells, have similar lipid constituents of gram-positive bacterial membranes, mainly PG component [9], high doses of daptomycin could attach to rhabdomyocytes lipid membranes and cause depolarization and cell lysis releasing the intracellular potassium content into the blood and increasing the serum levels of potassium before CPK serum level increases.

We hypothesize that elevated serum level of potassium can be an early sign of muscle breakage before the elevation of CPK level in the blood. We did observe a recurrent increase of potassium concentration on days 13, 15, 16, 19, 20, and 22, corresponding to days two, four, five, eight, 10, and a few hours following the start of daptomycin treatment as seen in Figure 2. Interestingly, although the last potassium peak was noted 10 days after daptomycin was initiated, the CPK serum level increased up to four-fold 24 hours and 400-fold 48 hours after the last recorded increase of potassium level which was on day 11 of daptomycin treatment. Daptomycin was later discontinued on day 26 after hospitalization, 15 days after the first dose. 

## Conclusions

Although daptomycin represents a good alternative for gram-positive resistant infections, its use is not without adverse effects. Reversible rhabdomyolysis is considered the one most associated with this antibiotic. Hyperkalemia, though not described as an adverse effect of daptomycin, could be related to its mechanism of action. Based on the description of our case, it would be important to consider monitoring serum potassium as an early sign of myopathy when high doses of daptomycin for prolonged times are planned to be given. 
